# Variable Ventilation Improved Respiratory System Mechanics and Ameliorated Pulmonary Damage in a Rat Model of Lung Ischemia-Reperfusion

**DOI:** 10.3389/fphys.2017.00257

**Published:** 2017-05-02

**Authors:** André Soluri-Martins, Lillian Moraes, Raquel S. Santos, Cintia L. Santos, Robert Huhle, Vera L. Capelozzi, Paolo Pelosi, Pedro L. Silva, Marcelo Gama de Abreu, Patricia R. M. Rocco

**Affiliations:** ^1^Laboratory of Pulmonary Investigation, Carlos Chagas Filho Institute of Biophysics, Federal University of Rio de JaneiroRio de Janeiro, Brazil; ^2^Pulmonary Engineering Group, Department of Anesthesiology and Intensive Care Therapy, University Hospital Carl Gustav Carus, Dresden University of TechnologyDresden, Germany; ^3^Department of Pathology, School of Medicine, University of São PauloSão Paulo, Brazil; ^4^Department of Surgical Sciences and Integrated Diagnostics, University of GenoaGenoa, Italy

**Keywords:** lung ischemia-reperfusion, variable ventilation, respiratory system mechanics, inflammation, molecular biology

## Abstract

Lung ischemia-reperfusion injury remains a major complication after lung transplantation. Variable ventilation (VV) has been shown to improve respiratory function and reduce pulmonary histological damage compared to protective volume-controlled ventilation (VCV) in different models of lung injury induced by endotoxin, surfactant depletion by saline lavage, and hydrochloric acid. However, no study has compared the biological impact of VV vs. VCV in lung ischemia-reperfusion injury, which has a complex pathophysiology different from that of other experimental models. Thirty-six animals were randomly assigned to one of two groups: (1) ischemia-reperfusion (IR), in which the left pulmonary hilum was completely occluded and released after 30 min; and (2) Sham, in which animals underwent the same surgical manipulation but without hilar clamping. Immediately after surgery, the left (IR-injured) and right (contralateral) lungs from 6 animals per group were removed, and served as non-ventilated group (NV) for molecular biology analysis. IR and Sham groups were further randomized to one of two ventilation strategies: VCV (*n* = 6/group) [tidal volume (V_T_) = 6 mL/kg, positive end-expiratory pressure (PEEP) = 2 cmH_2_O, fraction of inspired oxygen (FiO_2_) = 0.4]; or VV, which was applied on a breath-to-breath basis as a sequence of randomly generated V_T_ values (*n* = 1200; mean V_T_ = 6 mL/kg), with a 30% coefficient of variation. After 5 min of ventilation and at the end of a 2-h period (Final), respiratory system mechanics and arterial blood gases were measured. At Final, lungs were removed for histological and molecular biology analyses. Respiratory system elastance and alveolar collapse were lower in VCV than VV (mean ± SD, VCV 3.6 ± 1.3 cmH_2_0/ml and 2.0 ± 0.8 cmH_2_0/ml, *p* = 0.005; median [interquartile range], VCV 20.4% [7.9–33.1] and VV 5.4% [3.1–8.8], *p* = 0.04, respectively). In left lungs of IR animals, VCV increased the expression of interleukin-6 and intercellular adhesion molecule-1 compared to NV, with no significant differences between VV and NV. Compared to VCV, VV increased the expression of surfactant protein-D, suggesting protection from type II epithelial cell damage. In conclusion, in this experimental lung ischemia-reperfusion model, VV improved respiratory system elastance and reduced lung damage compared to VCV.

## Introduction

Ischemia-reperfusion (IR) injury remains a major problem after lung transplantation, and may result in severe lung damage with development of the acute respiratory distress syndrome in donor lungs or primary graft dysfunction in lung transplant recipients (Christie et al., 2005) Lung damage occurs early in the ischemic period, but is exacerbated during reperfusion (de Perrot et al., 2003). During ischemia, the anoxic condition, combined with a lack of mechanotransduction in the arterioles and capillaries (Lansman, 1988), induces dysfunction of endothelial and epithelial cells and other immune cells, as well as activation of nuclear factor-κB-derived cytokines (Ishiyama et al., 2005). These changes translate into increased pulmonary vascular resistance and alveolar edema, which impair lung function (Jurmann et al., 1990).

The protective mechanical ventilation strategies, which have been used during all stages of lung transplantation (Soluri-Martins et al., 2015), originated from studies in ARDS patients (Wiedemann et al., 2000); however, no clear recommendations are currently available (Meyer et al., 2014). Variable ventilation mimics the physiological fluctuation of tidal volume observed in resting subjects (Tobin et al., 1988; Frey et al., 1998), and compared to conventional protective, but non-variable mechanical ventilation, has been associated with better lung mechanics (Gama de Abreu et al., 2008; Spieth et al., 2009), reduced lung damage (Spieth et al., 2009; Kiss et al., 2016; Samary et al., 2016), and, ultimately, better mechanotransduction at the alveolar-capillary membrane level in experimental lung injury induced by surfactant depletion through saline lavage (Spieth et al., 2009), acid aspiration (Ma et al., 2011), and endotoxin (Samary et al., 2016). However, to date, no study has compared the biological impact of VV vs. conventional protective volume-controlled ventilation (VCV) mode in ARDS induced by lung ischemia-reperfusion. Lung ischemia-reperfusion (IR) injury is a complex phenomenon involving not only intracellular injury processes, but also injurious inflammatory responses and biochemical changes; thus, the pathophysiology of lung injury due to IR is different from that of the aforementioned ARDS models (den Hengst et al., 2010; Matute-Bello et al., 2011). Within this context, we hypothesized that VV might reduce early pulmonary inflammation and alveolar endothelial cell and type II epithelial cell injury, thus improving respiratory system mechanics and reducing lung damage. To assess this, we designed the present study to evaluate the effects of VV vs. VCV on lung function and histology, biological markers associated with inflammation, and damage inflicted to alveolar epithelial and endothelial cells in a rat model of lung ischemia-reperfusion injury.

## Materials and methods

### Ethics statement

This study was approved by the Animal Care Committee of the Health Sciences Center, Federal University of Rio de Janeiro (CEUA-019), and registered with the Brazilian National Council for Animal Experimentation Control. All animals received humane care in compliance with the “Principles of Laboratory Animal Care” formulated by the National Society for Medical Research and the U.S. National Academy of Sciences *Guide for the Care and Use of Laboratory Animals*.

### Animal preparation

Thirty-six Wistar rats (weight 526 ± 117 g) were premedicated with diazepam (10 mg/kg, Cristália, Itapira, Brazil), midazolam (2 mg/kg, União Química, São Paulo, Brazil), and ketamine (50–100 mg/kg, Cristália, Itapira, Brazil) intraperitoneally. An intravenous catheter (Jelco 24G, Becton, Dickinson and Company, New Jersey, USA) was inserted into the tail vein and animals anesthetized with midazolam (2 mg/kg) and ketamine (50 mg/kg/h), while Ringer's lactate (7 mL/kg/h, B.Braun, Crissier, Switzerland) was infused continuously. Anesthetized animals were kept in the dorsal recumbent position and tracheotomized via a midline neck incision after subcutaneous injection of 2% lidocaine (Cristália, Itapira, SP, Brazil). The right internal carotid artery was cannulated for blood sampling and measurement of mean arterial pressure (MAP). A solution containing Ringer's lactate and 8% albumin (Cristália, Itapira, Brazil) was administered in 0.5-mL increments to keep MAP ≥ 60 mmHg. Heart rate, MAP, and rectal temperature were recorded continuously (Networked Multiparameter Veterinary Monitor LifeWindow 6000V, Digicare Animal Health, Florida, USA). Body temperature was maintained at 37.5 ± 1°C using a heating bed. Animals were then paralyzed (pancuronium bromide, 2 mg/kg intravenously) and mechanically ventilated (Inspira, Harvard Apparatus, Holliston, MA, USA) in VCV mode with V_*T*_ = 6 mL/kg, respiratory rate (RR) set to maintain pHa = 7.35–7.45, Fio_2_ = 0.4, and positive end-expiratory pressure (PEEP) = 2 cmH_2_O.

### Experimental protocol

**Figure 1:**
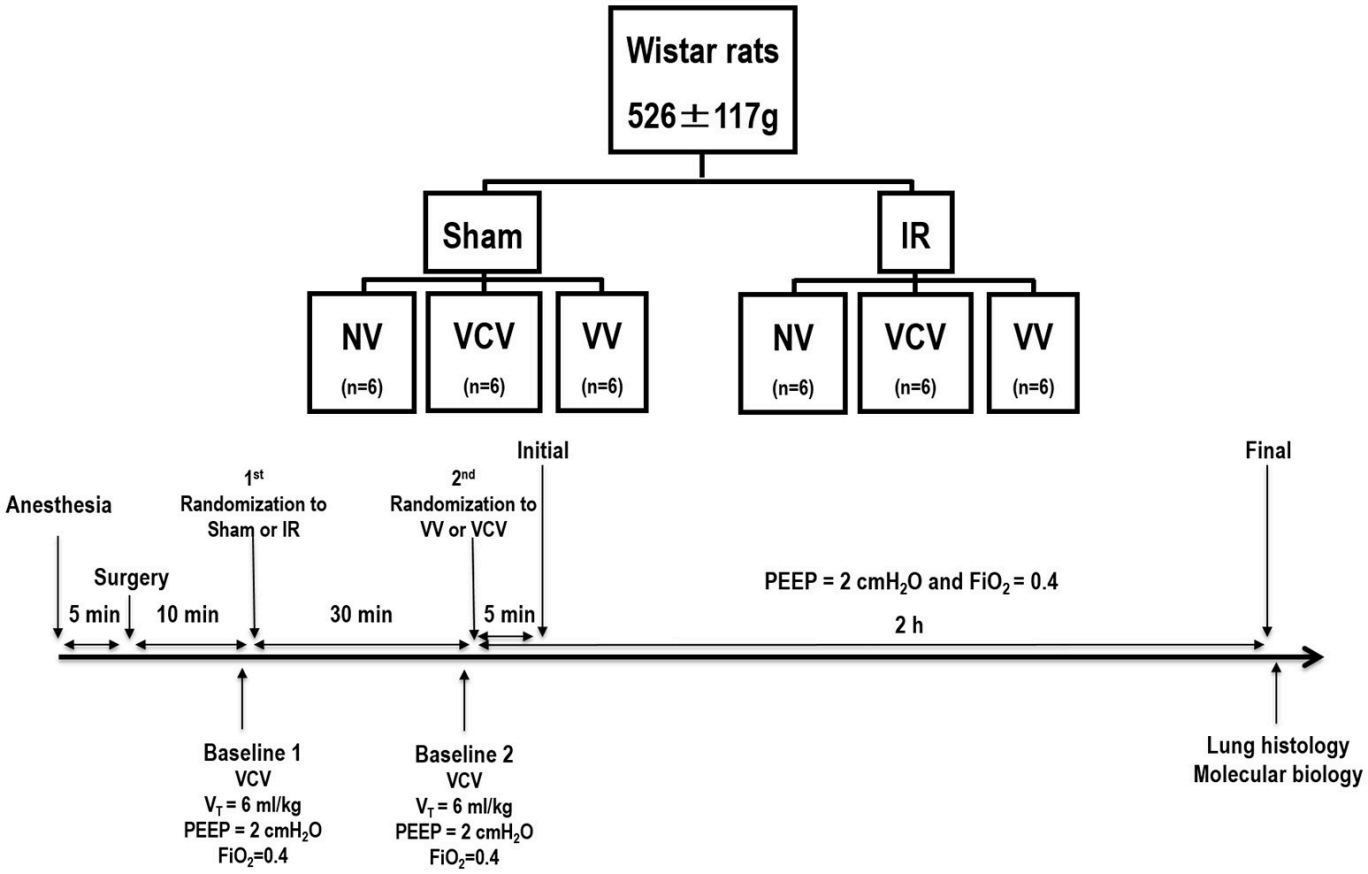
**Timeline representation of the experimental protocol**. Baseline 1: evaluation of baseline lung health; Baseline 2: evaluation of lung damage induced by left pulmonary hilum clamping (ischemia-reperfusion) compared to sham surgery. Initial: Immediately after randomization to variable controlled ventilation (VV) or volume controlled ventilation (VCV). NV: non-ventilated animals. V_*T*_: tidal volume; PEEP: positive-end expiratory pressure. FiO_2_: fraction of inspired oxygen. Gas exchange and lung mechanics were evaluated at Baseline 1, Baseline 2, Initial, and Final.

After hemodynamic stabilization, respiratory system mechanics, and arterial blood-gases (Radiometer ABL80 FLEX, Copenhagen, Denmark) were measured (Baseline 1; Figure 1). Five minutes later, the skin was incised and a median sternotomy was performed. An eyelid retractor was used to keep the sternum open. Animals were randomly assigned to one of two groups: (1) ischemia-reperfusion (IR), in which the left pulmonary hilum was completely occluded using a small metallic clamp, and (2) Sham, in which animals underwent the same surgical manipulation but without hilar clamping. After 30 min, the clamp was gradually released within 10 s in IR group and respiratory system mechanics and arterial blood-gases were measured again in both groups (Baseline 2). Immediately after surgery, the left (IR injury) and right (contralateral) lungs from 6 animals in each group were removed, and served as a non-ventilated (NV) group for molecular biology analysis. The IR and Sham groups were further randomized to one of two ventilation strategies: conventional protective volume-controlled ventilation (VCV, *n* = 6/group) [tidal volume (V_T_) = 6 mL/kg, RR set to maintain pHa = 7.35–7.45, positive end-expiratory pressure (PEEP) = 2 cmH_2_O, fraction of inspired oxygen (FiO_2_) = 0.4] (IR-VCV and Sham-VCV) and variable ventilation (VV), which was applied on a breath-to-breath basis as a sequence of randomly generated V_*T*_ values (Gaussian distribution, *n* = 1,200; mean V_T_ = 6 mL/kg), with a 30% coefficient of variation (Supplemental Figure 1; nVentInspira, Dresden, Germany; Huhle et al., 2014; IR-VV and Sham-VV). After 5 min of ventilation (Initial) and at the end of a 2-h period (Final), respiratory system mechanics and arterial blood-gases were measured. Animals were then killed by an overdose of intravenous sodium thiopental (50 mg/kg, Cristália, Itapira, Brazil) and their lungs extracted for histological and molecular biology analyses.

An additional 12 animals—6 not subjected to the IR procedure (Healthy) and 6 exposed to IR—were used for model characterization (Supplemental Figure 2). In short, a total of 48 animals were used in this study.

### Data acquisition and respiratory system mechanics

Airflow, volume, and airway pressure (P_aw_) were recorded with a computer running custom software written in LabVIEW® (National Instruments, Texas, USA; Silva et al., 2013). All signals were amplified (TAM-D HSE Plugsys Transducers Amplifiers, Module Type 705/2, Harvard Apparatus, MA, USA) and sampled at 200 Hz with a 12-bit analog-to-digital converter (National Instruments, Texas, USA). Respiratory system elastance (E,_*RS*_) and resistance (R,_*RS*_) were calculated based on the equation of motion (Uhlig et al., 2014): $P_{aw}\left(t\right)=R_{RS}•\overset{.\cdot }{V}\left(t\right)+E_{RS}•V\left(t\right)+P_{0}$, where *t* is time, *V* is volume, and *P*_0_ is *P*_*aw*_ at end-expiration. Volume-independent elastance (E1,_RS_) and volume-dependent elastance (E2,_RS_) as well as the E,_RS_ non-linearity index (%E2) were calculated cycle-by-cycle as described elsewhere (Carvalho et al., 2013b).

### Histology

Lung histology (light and electron microscopy) was evaluated in left (IR-injury) and right (contralateral) lungs in all groups.

### Light microscopy

A laparotomy was performed immediately after determination of respiratory system mechanics, and heparin (1,000 IU) was injected into the vena cava. The trachea was clamped at end-expiration (PEEP = 2 cmH_2_O) and the abdominal aorta and vena cava were sectioned, yielding a massive hemorrhage that quickly killed the animals. Immediately after removal, the left lung was flash-frozen by immersion in liquid nitrogen), fixed in Carnoy's solution, and paraffin-embedded. Sections (4 μm thick) were cut and stained with hematoxylin-eosin. Morphometric analysis was done using an integrating eyepiece with a coherent system made of a 100-point grid consisting of 50 lines of known length, coupled to a conventional light microscope (Axioplan, Zeiss, Oberkochen, Germany). The volume fraction of collapsed and normal pulmonary areas were determined by the point-counting technique (Weibel, 1990), at a magnification of × 200 across 10 random, non-coincident microscopic fields. Briefly, points falling on collapsed or normal pulmonary areas were counted and divided by the total number of points in each microscopic field.

### Transmission electron microscopy

Three slices (2 × 2 × 2 mm) were cut from three different segments of the left and right lungs (apex, middle, and base of the lung). They were then fixed in 2.5% glutaraldehyde and phosphate buffer, 0.1 M (pH = 7.4) for electron microscopy analysis (JEOL 1010 Transmission Electron Microscope; Japan Electron Optics Laboratory Co, Tokyo, Japan). On each electron microscopy image (20 fields/animal), damage to type II epithelial and endothelial cells was graded on a five-point, semi-quantitative, severity-based scoring system as follows: 0 = normal lung parenchyma; 1 = changes in 1–25%; 2 = 26–50%, 3 = 51–75%, and 4 = 76–100% of examined tissue (Samary et al., 2015). Investigators (RSS and VLC) blinded to the origin of the material performed the microscopic examination. Agreement between observers was good (*k* = 0.73).

### Biological markers of inflammation and epithelial and endothelial cell damage

Quantitative real-time reverse transcription polymerase chain reaction (RT-PCR) was performed to assess biological markers of inflammation (interleukin [IL]-6), oxidative stress (nuclear factor erythroid 2-derived factor-2 [Nrf2]), epithelial cell damage (surfactant protein [SP]-D), and endothelial cell damage (angiopoietin [Ang]-1, Ang-2, receptor tyrosine kinase of Tie family, and intercellular adhesion molecule [ICAM]-1). Central slices of the left and right lungs were cut, collected in cryotubes, flash-frozen in liquid nitrogen, and stored at −80°C. Total RNA was extracted (RNeasy Plus Mini Kit [Qiagen, Hilden, Germany]), and RNA concentration measured by spectrophotometry (Nanodrop ND-1000 system [ThermoScientific, Wilmington, DE, USA]). First-strand cDNA was synthesized from total RNA (Quantitec reverse transcription kit [Qiagen]). Primers are described in Supplemental Table 1. Relative mRNA levels were measured with a SYBR green detection system in an ABI 7500 real-time PCR analyzer (Applied Biosystems, Foster City, CA, USA). Samples were run in triplicate. For each sample, the expression of each gene was normalized to the acidic ribosomal phosphoprotein P0 (*36B4*) housekeeping gene (Akamine et al., 2007) and expressed as fold change relative to non-ventilated (NV) animals, respectively for the left (IR injury) and right (contralateral) lungs, using the 2^−ΔΔCt^ method.

### Statistical analysis

Sample size calculation was based on pilot studies and on a previous report in healthy rodents using similar ventilator settings (Henriques et al., 2016). A sample size of six animals per group would provide the appropriate power (1–β = 0.8) to identify significant (α = 0.05) differences in E,_RS_ between VCV and VV, taking into account an effect size *d* = 1.85, a two-sided test, and a sample size ratio of 1 (G^*^Power 3.1.9.2, University of Düsseldorf, Düsseldorf, Germany).

Respiratory system mechanics and blood-gases between Baseline 1 and Baseline 2 were compared by a paired *t*-test. Comparisons among groups over time were done by two-way repeated-measures ANOVA followed by Bonferroni's test, as were lung histological analyses. Molecular biology analyses were performed using the Kruskal–Wallis test followed by Dunn's test in specimens from the left (IR injury) and right (contralateral) lungs. Parametric data were expressed as mean ± standard deviation (*SD*) and non-parametric data as median (interquartile range). All tests were performed using the GraphPad Prism v6.01 statistical software package (GraphPad Software, La Jolla, CA, USA).

## Results

### Ischemia-reperfusion injury protocol

V_T_, CV of V_T_, RR, pHa, PaO_2_/FiO_2_, PaCO_2_, and ${\text{HCO}}_{3}^{-}$ did not differ between Baselines 1 and 2 (Supplemental Table 2). Conversely, in IR group, E,_RS_ and Paw during the time course of the experiment (Baseline 1 vs. Baseline 2), mainly due to E2,_RS_. Accordingly, %E2 was increased in IR compared to Sham animals (*p* = 0.007). After injury, IL-6 and Nrf2 expressions were increased in left compared to right lungs. Ang-2 was reduced in both left and right lungs compared to NV (Supplemental Figure 1).

### Comparison between variable and conventional volume-controlled ventilation

V_T_, RR, and ${\text{HCO}}_{3}^{-}$ did not differ between Initial and Final (Table 1). CV of V_T_ increased, while E,_RS_ and P_aw_ decreased in VV compared to VCV, in both Sham and IR animals. Compared to VCV, VV resulted in lower E1,_RS_ in Sham and reduced E2,_RS_ in IR animals. VV improved PaO_2_/FiO_2_ in Sham and decreased PaCO_2_ in IR. VV, compared to VCV, also decreased pHa both in Sham and in IR animals. Cumulative fluid volume infused showed a significant time effect, without differences among groups.

**Table 1:** **Respiratory parameters during mechanical ventilation**.

**Parameter**	**Sham or IR**	**VCV or VV**	**Initial**	**Final**	**Time Effect**	**Group Effect**	**Time vs. Group Effect**
V_T_ (mL/kg)					*P* = 0.85	*P* = 0.87	*P* = 0.84
	Sham	VCV	6.9 ± 2.4	5.9 ± 0.3			
		VV	6.0 ± 0.3	6.0 ± 0.4			
	IR	VCV	6.1 ± 0.6	6.1 ± 0.6			
		VV	6.0 ± 0.1	6.0 ± 0.3			
CV of V_T_ (%)					*P* < 0.001	*P* < 0.001	*P* < 0.001
	Sham	VCV	1.9 ± 1.1	1.9 ± 0.9			
		VV	1.3 ± 0.2	30.1 ± 2.0	^***^	^###^	
	IR	VCV	1.3 ± 0.4	1.8 ± 0.7			
		VV	1.3 ± 0.3	29.9 ± 0.8	^***^	^###^	
E._RS_ (cmH_2_O/mL)					*P* = 0.003	*P* = 0.005	*P* = 0.0002
	Sham	VCV	2.9 ± 0.6	3.7 ± 0.9	^**^		
		VV	2.2 ± 0.5	1.8 ± 0.5		^###^	
	IR	VCV	2.5 ± 0.9	3.6 ± 1.3	^***^		
		VV	2.1 ± 0.3	2.0 ± 0.8		^##^	
E1._RS_ (cmH_2_O/mL)					*P* = 0.007	*P* = 0.006	*P* = 0.005
	Sham	VCV	2.3 ± 0.5	2.7 ± 0.6	^***^		
		VV	1.7 ± 0.5	1.6 ± 0.5		^###^	
	IR	VCV	1.9 ± 0.4	2.3 ± 0.5	^**^		
		VV	1.7 ± 0.3	1.6 ± 0.2			
E2._RS_ (cmH_2_O/mL)					*P* = 0.01	*P* = 0.11	*P* = 0.02
	Sham	VCV	0.3 ± 0.2	0.6 ± 0.4			
		VV	0.2 ± 0.1	0.1 ± 0.1			
	IR	VCV	0.3 ± 0.5	0.7 ± 0.3	^**^		
		VV	0.2 ± 0.1	0.2 ± 0.1		^#^	
%E2 (%)					*P* = 0.13	*P* = 0.53	*P* = 0.007
	Sham	VCV	22.5 ± 9.9	31 ± 11.7			
		VV	23.3 ± 21.2	12.7 ± 21.9			
	IR	VCV	19.2 ± 23.0	40.6 ± 9.3	^**^		
		VV	25.7 ± 9.2	23.7 ± 7.0			
P_aw_ (cmH_2_O)					*P* < 0.001	*P* = 0.003	*P* < 0.001
	Sham	VCV	11.7 ± 1.8	14.6 ± 1.6	^***^		
		VV	11.3 ± 1.6	10.0 ± 1.2	^*^	^###^	
	IR	VCV	11.3 ± 1.6	14.7 ± 1.9	^***^		
		VV	11.5 ± 0.9	11.0 ± 0.7		^###^	
RR (min^−1^)					*P* = 0.09	*P* = 0.67	*P* = 0.86
	Sham	VCV	43 ± 10	42 ± 9			
		VV	39 ± 6	39 ± 6			
	IR	VCV	45 ± 11	44 ± 11			
		VV	40 ± 4	40 ± 5			
pHa					*P* = 0.005	*P* = 0.002	*P* = 0.14
	Sham	VCV	7.35 ± 0.05	7.30 ± 0.06	^*^		
		VV	7.42 ± 0.02	7.39 ± 0.03		^#^	
	IR	VCV	7.37 ± 0.05	7.34 ± 0.03			
		VV	7.40 ± 0.04	7.41 ± 0.04		^#^	
PaO_2_/FiO_2_					*P* = 0.02	*P* = 0.16	*P* = 0.04
	Sham	VCV	322 ± 44	339 ± 38			
		VV	329 ± 60	416 ± 28	^*^		
	IR	VCV	296 ± 56	338 ± 83			
		VV	375 ± 58	350 ± 58			
PaCO_2_(mmHg)					*P* = 0.92	*P* = 0.004	*P* = 0.15
	Sham	VCV	36 ± 5	42 ± 7			
		VV	34 ± 2	36 ± 2			
	IR	VCV	35 ± 4	37 ± 5			
		VV	31 ± 2	28 ± 5		^#^	
HCO_3_(mEq/L)					*P* = 0.12	*P* = 0.04	*P* = 0.61
	Sham	VCV	20 ± 1	20 ± 2			
		VV	21 ± 2	20 ± 3			
	IR	VCV	22 ± 1	20 ± 2			
		VV	21 ± 2	20 ± 3			
Fluids(mL)					*P* < 0.0001	*P* = 0.23	*P* = 0.46
	Sham	VCV	2.5 [1.5-3.0]	7.5 [3.25-79.6]	^**^		
		VV	2.5 [0.4-3.6]	5.75 [3.1-7.5]			
	IR	VCV	1.5 [0.4-2.25]	2.25 [0.4-6.0]	^*^		
		VV	2.0 [0.75-2.9]	5.0 [3.75-8.75]	^*^		

*Respiratory parameters at Initial and End. Sham: animals subjected to surgical manipulation alone, without clamping the left pulmonary hilum; IR: animals subjected to surgical manipulation and ischemia-reperfusion injury by clamping the left pulmonary hilum. V_T_, Tidal volume; CV of V_T_, coefficient of variation of tidal volume; E,_RS_, respiratory system elastance; E1,_RS_: volume-independent elastance; E2,_RS_, volume-dependent elastance; %E2, E,_RS_ non-linearity index; P_aw_, airway pressure; pHa, arterial pH; PaO_2_/FiO_2_, arterial oxygen partial pressure divided by oxygen fraction; PaCO_2_, arterial carbon dioxide partial pressure; HCO_3_, bicarbonate; Fluids, cumulative fluids infused during 2 h of mechanical ventilation. Values are mean ± standard deviation (SD) of 6 animals or median [interquartile range] in each group. Comparisons were done using two-way repeated-measures ANOVA followed by Holm-Šidák's multiple comparisons test (p < 0.05)*.

* *p < 0.05*,

** *p < 0.01*,

*** *p < 0.001 vs. Initial*;

# *p < 0.05*,

## *p < 0.01*,

### *p < 0.001 vs. respective VCV*.

**Figure 2:**
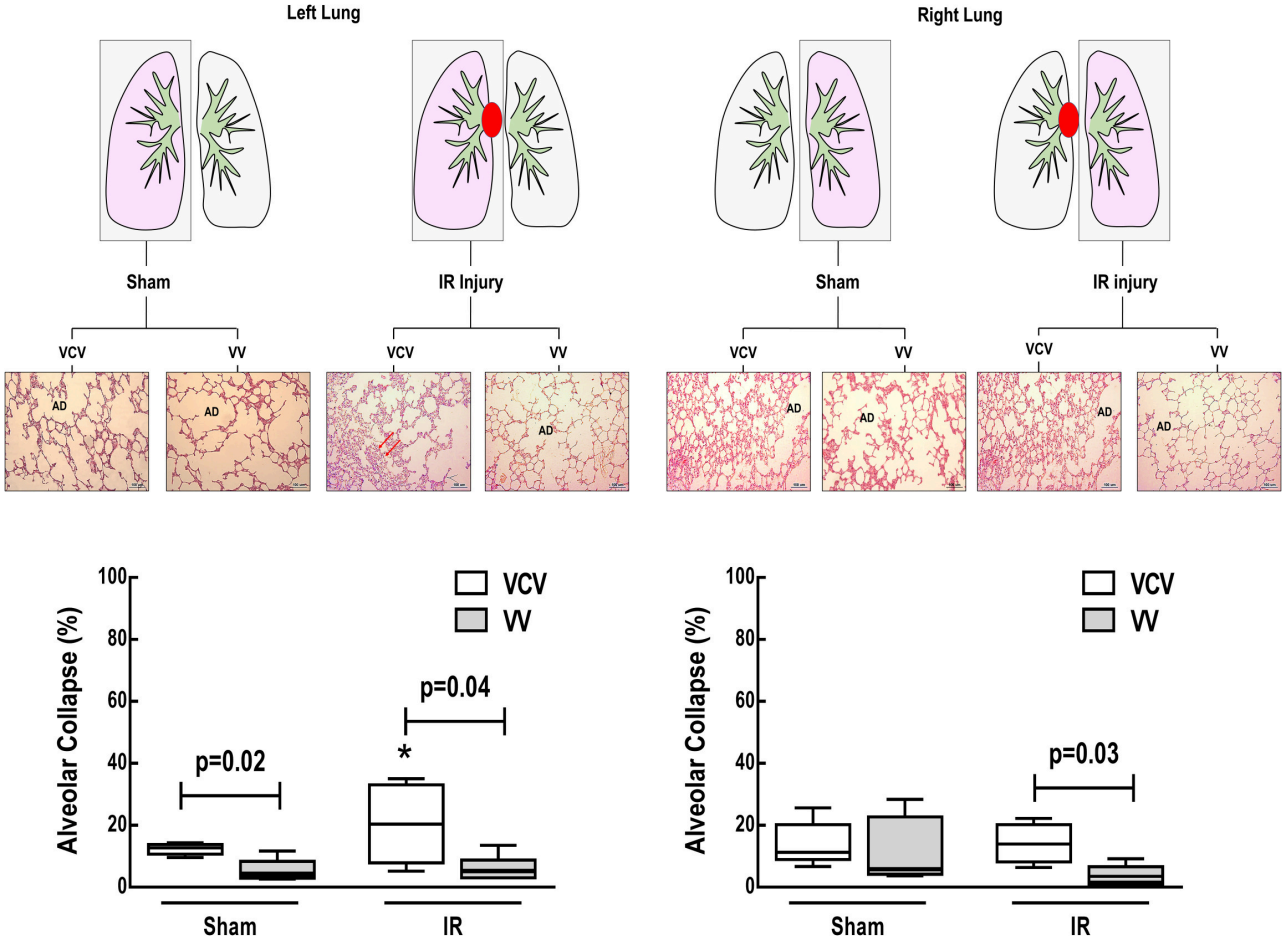
**Schematic representation of lung ischemia-reperfusion (IR) injury and sham clamping in left (injured) and right (contralateral) lungs, in animals ventilated in volume-controlled (VCV) or variable controlled ventilation (VV)**. AD: Alveolar duct. Photomicrographs of lung parenchyma stained with hematoxylin-eosin, original magnification x200. Arrows: alveolar collapse. Fraction areas of alveolar collapse in left and right lungs of Sham and IR animals ventilated with VCV and VV. Values represent medians and whiskers represent the 10–90 percentile range of 6 animals in each group. Two-way repeated-measured ANOVA followed by Bonferroni's *post-hoc* test (*p* < 0.05). ^*^Significantly different from Sham group (*p* < 0.05).

**Figure 3:**
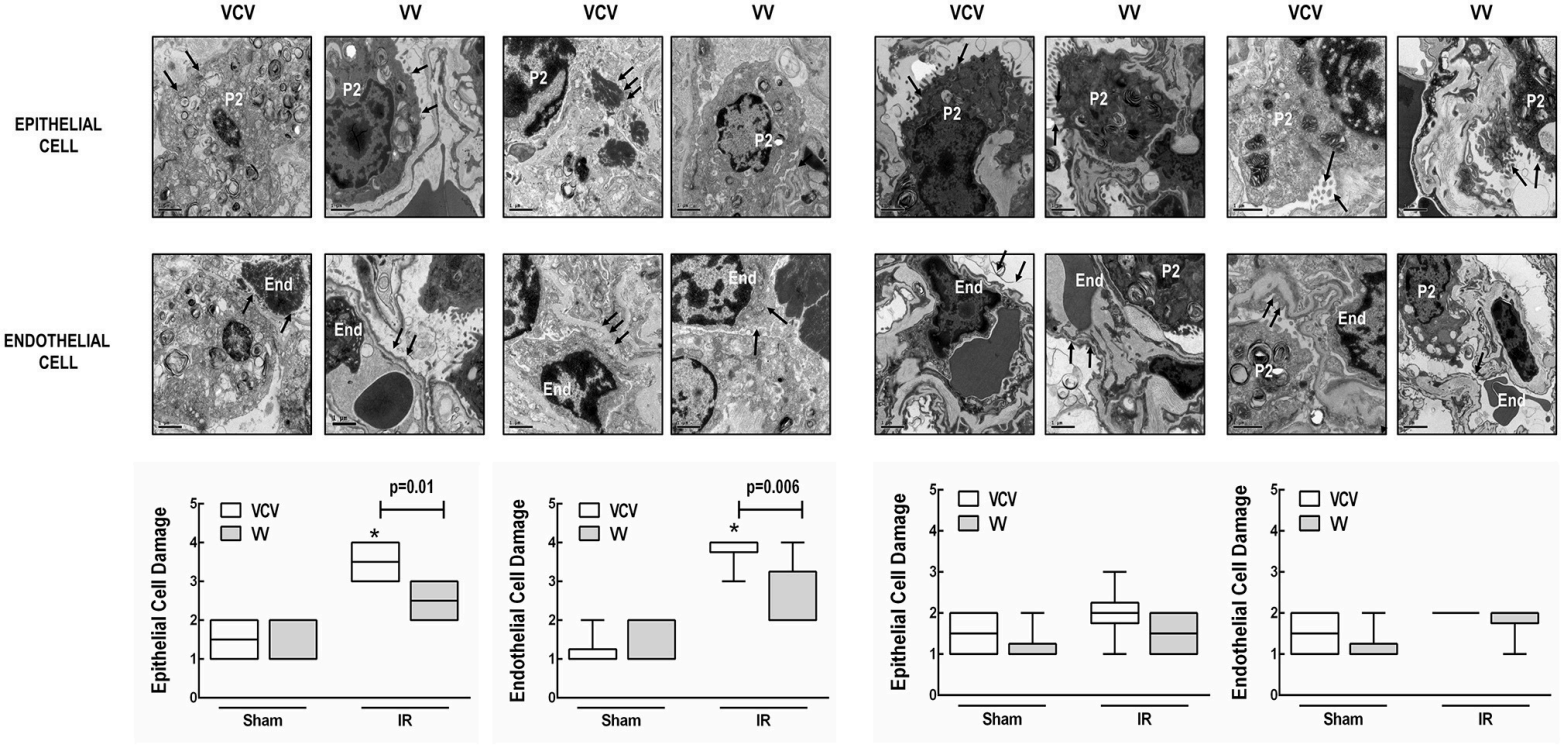
**Electron microscopy of lung ischemia-reperfusion (IR) injury and sham clamping in left (injured) and right (contralateral) lungs, in animals ventilated in volume-controlled (VCV) or variable controlled ventilation (VV)**. Photomicrographs are representative of epithelial (type 2 epithelial cell, P2) and endothelial (End) cell damage (arrows) obtained from lung sections derived from 6 animals. Lung injury score (epithelial and endothelial cell damage) assessed by electron microscopy. Values represent medians and whiskers represent the 10–90 percentile range of 6 animals in each group. Two-way repeated-measures ANOVA followed by Bonferroni's *post-hoc* test (*p* < 0.05). ^*^Significantly different from Sham group (*p* < 0.05).

In VCV, alveolar collapse and epithelial and endothelial cell damage were higher in IR than Sham in left lungs (Figures 2, 3). However, these parameters did not differ in right lungs.

In left lungs, alveolar collapse was lower with VV than VCV in both Sham and IR groups. In right (contralateral) lungs, VV reduced alveolar collapse compared to VCV only in animals that underwent IR injury of the left lung (Figure 2). Ultrastructural evidence of epithelial and endothelial damage was lower after VV than VCV in IR-injured lungs (Figure 3).

**Figure 4:**
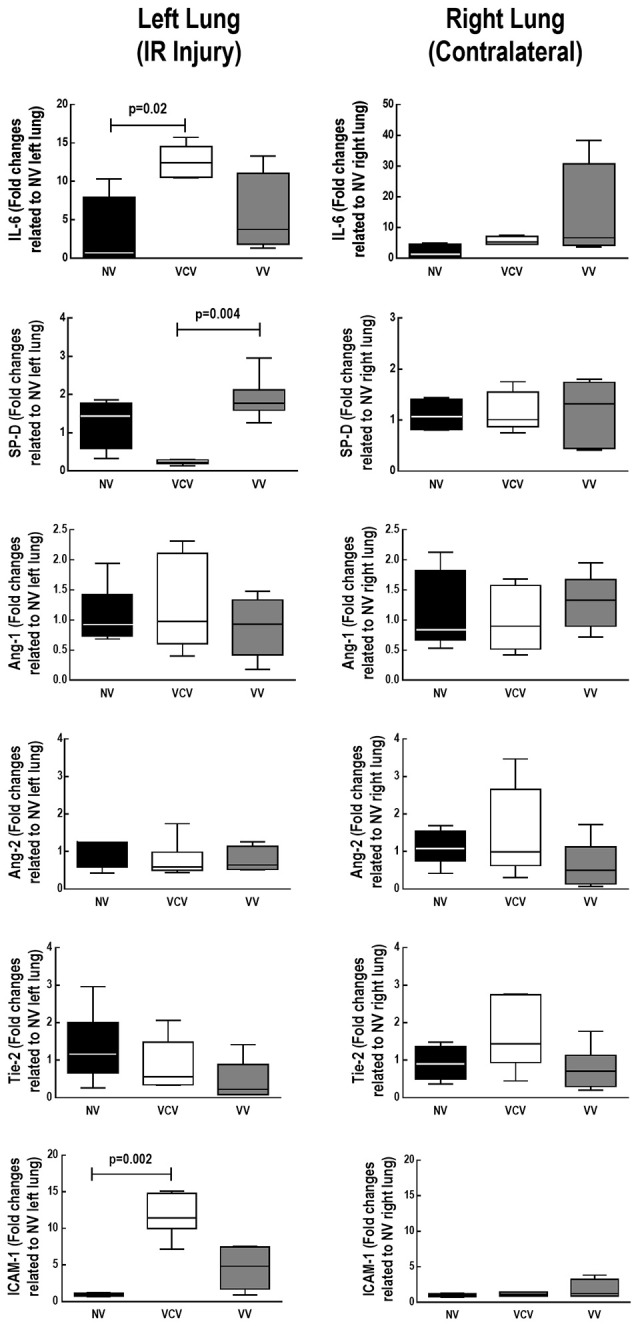
**Real-time polymerase chain reaction analysis of biological markers associated with inflammation [interleukin (IL)-6]; type II epithelial cell damage [surfactant protein (SP)-D]; and endothelial cell damage [angiopoietin (Ang) 1 and 2, receptor tyrosine kinase of Tie family (Tie), and intercellular adhesion molecular (ICAM)-1]**. VCV: volume controlled ventilation; VV: variable controlled ventilation. Left lung: ischemia-reperfusion injury; right lung: contralateral. Values represent medians and whiskers represent the 10–90 percentile range of 6 animals in each group. Relative gene expression was calculated as a ratio of the average gene expression levels compared with the reference gene (*36B4*) and expressed as fold change relative to non-ventilated (NV) left lung or NV right lung.

VCV, but not VV, increased the expression of IL-6 and ICAM-1. VV increased the expression of SP-D compared to VCV (Figure 4).

## Discussion

In the rat model of lung ischemia-reperfusion used herein, VV, compared to VCV: (1) decreased E,_RS_ mainly due to lower E2,_RS_; (2) was associated with less alveolar collapse; and (3) increased SP-D expression. In addition, VCV, but not VV, increased expression of IL-6 and ICAM-1 in the left lung after IR injury.

### Ischemia-reperfusion injury model

It has been suggested that the initial phase (30 min) of IR is more dependent on products from activated pulmonary macrophages, such as IL-6 and tumor necrosis factor-α (den Hengst et al., 2010). Additionally, a burst of reactive oxygen species (ROS) appears immediately after reperfusion within hypoxic endothelial cells. These cells, in turn, overwhelm the ROS by synthetizing antioxidant defenses through Nrf2 transcription, which protects against oxidative tissue injury (Nguyen et al., 2009). In our study, after 30 min of left pulmonary hilum occlusion, IL-6 and Nrf2 expressions increased in left (IR-injured) compared to right (contralateral) lungs (Supplemental Figure 1), followed by impairment in lung mechanics. We may infer that 30 min of ischemia lead to oxidative stress stimulation (Nrf2), which, in turn, activates alveolar macrophages to release proinflammatory cytokines (IL-6). Nrf2 is a transcription factor that regulates the expression of antioxidant proteins, thus protecting against the oxidative stress damage associated with ischemia-reperfusion injury (Nguyen et al., 2009). On the other hand, IL-6 is an important mediator that induces and perpetuates lung inflammation in several conditions (Mittal et al., 2014). Ultimately, these early biomarkers may yield increased atelectasis, thus augmenting E,_RS_.

Previous experimental studies in ARDS models induced by surfactant depletion through saline lavage (Spieth et al., 2009), acid aspiration (Ma et al., 2011), and endotoxin (Samary et al., 2016) have shown that VV improves gas exchange and respiratory mechanics, as well as reduces lung damage and inflammation. However, the pathophysiology of lung IR is complex, with multiple molecular and cellular mechanisms involved (den Hengst et al., 2010), thus differing from the pathophysiology and morphological changes present in other experimental models of ARDS (den Hengst et al., 2010); this is likely to result in differing biological responses to mechanical ventilation strategies. In this context, our group recently reported, in a model of endotoxin-induced pulmonary and extrapulmonary ARDS, that VV improved lung function in both etiologies (Samary et al., 2016). Nevertheless, the beneficial effects of VV on biological markers were more pronounced in pulmonary ARDS than in extrapulmonary ARDS. In short, depending on ARDS etiology, VV may be or not beneficial. Additionally, lung morphological changes differ according to whether extrapulmonary ARDS was induced by intraperitoneal administration of endotoxin or after lung IR. Specifically, lung IR is characterized by (1) vascular damage, which contributes to the development of pulmonary edema; (2) type II epithelial cell dysfunction with impairment of surfactant composition and production; and (3) preserved type 1 epithelial cell function (den Hengst et al., 2010). In contrast, when endotoxin is injected intraperitoneally, endothelial cell damage is observed predominantly, without significant damage to type I or II epithelial cells over a 24-h period (Menezes et al., 2005; Riva et al., 2008).

To the best of our knowledge, the present study was the first to investigate the effects of VV and VCV on lung morphofunction and biological markers associated with VILI in experimental ARDS induced by lung ischemia-reperfusion. To minimize the potential effects of VV and VCV, a protective tidal volume was used during both strategies.

### Effects of VV on respiratory function

The ischemia period was likely followed by loss of lung volume, which, in turn, can cause closure of peripheral airways during expiration during low-V_T_ ventilation. Failure to reopen these airways during inspiration may lead to atelectasis, with consequent deterioration of respiratory mechanics. VV may act, in this scenario, by promoting lung recruitment through stochastic resonance; consequently, the driving pressure for a given V_T_ would be theoretically reduced (Huhle et al., 2016). After 2 h, we observed E,_RS_ reductions in both Sham and IR animals, and, in the latter case, was followed by a decrement in E2,_RS_ (Table 1). The reduction in E2,_RS_ is in line with low tidal recruitment/overdistension (Kano et al., 1994; Carvalho et al., 2013a), which denotes better V_T_ distribution across the lungs. Interestingly, similar behavior was not observed in E1,_RS_, because this parameter is independent of changes in tidal volume. Oxygenation improved during VV in the Sham group. During VV, improvement in gas-exchange is usually a consequence of enhanced ventilation-perfusion matching (Spieth et al., 2009). However, we did not observe such oxygenation improvement in IR animals after VV. This can be explained by: (1) severe ventilation-perfusion mismatch, as usually found in ischemia-reperfusion injury models (Löckinger et al., 2001); (2) even in unilateral ischemia, physiological changes also appear on the contralateral non-ischemic lung, suggesting that injurious signals are humorally secreted as well (Palazzo et al., 1992), and, as a consequence, overall oxygenation may not change.

### Effects of VV in IR-injured lungs

In left lungs subjected or not to ischemia (Sham), we observed a decrease in alveolar collapse after VV implementation, because of lung recruitment and better V_T_ distribution across alveolar units. Although there is no classification scheme for histological changes after ischemia-reperfusion, it is well accepted that endothelial cells may become apoptotic, as demonstrated by cellular rounding and contraction (Gilmont et al., 1996). Moreover, hypoxia and impairment of mechanotransduction induce upregulation of cell surface adhesion molecules, such as ICAM-1, in endothelial cells (Fisher et al., 1991; Ishiyama et al., 2005). In the present study, IL-6 and ICAM-1 increased with VCV, but not VV, which supports the hypothesis of further injury to the endothelial cells, due to inflammation, after ischemic injury with VCV. On the epithelial side of the lung, the imbalance of surfactant function plays an important role during lung ischemia-reperfusion in lung transplant recipients (Novick et al., 1996). The increase in SP-D expression in IR animals ventilated with VV suggests surfactant synthesis. These results are explained by the fact that endothelial cells respond in a different way to uniaxial cyclic stretch (Suzuki et al., 1997) and to variable cell stretch (Imsirovic et al., 2013), with the latter being associated with better cell function (adhesion), while epithelial cells are highly influenced by the application of variable stretch, which enhances either surfactant synthesis (Arold et al., 2009) or production/release (Arold et al., 2003; Thammanomai et al., 2013).

### Effects of VV ventilation in contralateral lungs

It has been shown that ischemia-reperfusion injury of one lung can lead to similar, but less severe, injury in the contralateral lung (Palazzo et al., 1992). In our model, since both perfusion and ventilation were blocked in the left lung, the right lung received all ventilation (~12 mL/kg) and perfusion (~300 mL/min/kg) for 30 min. Even under these supraphysiological conditions, no changes in IL-6 or Nrf2 mRNA expressions were found. Nevertheless, it has been suggested that most of the non-ischemic lung injury develops only following reperfusion of the ischemic lung, with red blood cell accumulation by chemotactic signaling (Wolf et al., 2009), after 30 min of reperfusion (Eppinger et al., 1997) in the contralateral lung. However, after 2 h of reperfusion, we did not observe major changes in mRNA expressions in the contralateral lung, whether in VV or VCV animals. The most relevant beneficial effect was the reduction in alveolar collapse after VV, but not VCV, as previously explained by the repercussions of VV on lung function. We may explain these results by comparing the ischemia models used in previous studies (Palazzo et al., 1992; Eppinger et al., 1997; Wolf et al., 2009) to the one employed herein. In addition, the complete abolition of ventilation-perfusion better reflects the surgical procedure used in lung transplantation (Boasquevisque et al., 2009). Although this is a negative result in relation to the expected effects of VV, we believe this is an important finding, as it is in line with the principle of “first do no harm.” During human lung transplantation, the contralateral lung is usually the native (non-transplanted) lung, which must be in a protective form of ventilation during and after the surgical procedure. Previous studies of single-lung ventilation have shown better outcomes when using low V_*T*_ (5–6 mL/kg) and moderate PEEP (5 cmH_2_O; Michelet et al., 2006; Yang et al., 2011; Shen et al., 2013), but not with higher PEEP levels (9 cmH_2_O), which were associated with decreased oxygenation, suggesting that PEEP>5 cmH_2_O may be not tolerated during or after single-lung ventilation (Rozé et al., 2012). This is of particular interest in VV, since, by changing V_T_, this mode can reduce driving pressure and maintain lung volume, which fundamentally differs from the incremental effects of PEEP.

### Clinical implications

Thoracic surgery procedures such as cardiopulmonary bypass and lung transplantation have evolved over the last two decades. Although ventilation strategies have shown limited progress compared with patient management practices (Soluri-Martins et al., 2015), evidence has emerged in favor of some specific ventilation strategies, depending on the stage of the lung transplantation process. Protective ventilatory strategies should be adopted in donors even in the absence of lung injury, with the application of low V_T_, recruitment maneuvers to maintain lung volume, and adequate PEEP. In the present study, we suggested that VV could be a valuable add-on in IR conditions.

### Limitations

This study has several limitations. First, one could argue that variable mechanical ventilation would be equivalent to regular ventilation with intermittent sighs. A sigh is defined as an increase in total lung volume above average values for only a few seconds, and can be observed in healthy, spontaneously breathing subjects. Sighs have been used to counteract progressive lung derecruitment during both controlled and assisted mechanical ventilation, showing moderate success for improving lung mechanics and gas exchange in acute lung injury, for example (Patroniti et al., 2002; Pelosi et al., 2003). During variable mechanical ventilation, sigh-like tidal volumes occur at intervals. Using a porcine model of lung atelectasis, Mutch et al. showed that variable controlled mechanical ventilation was superior to conventional ventilation combined with intermittent sighs to improve gas exchange and respiratory system compliance (Mutch et al., 2000). In experimental acute lung injury, Gama de Abreu et al. found that the variability of tidal volumes was higher with noisy PSV than with PSV and sighs (19.1 vs. 7.8%, median values). Accordingly, noisy PSV resulted in better oxygenation and reduced venous admixture than PSV with sighs (Gama de Abreu et al., 2008). In addition, Thammanomai et al. showed that variable mechanical ventilation, as compared to regular ventilation with periodic sighs, improves the inflammatory lung response of mice with acute lung injury (Thammanomai et al., 2008). These data suggest that the complex pattern of breathing during variable ventilation cannot be considered equivalent to the use of periodic intermittent sighs. Second, the ischemia period was set at 30 min, and caution is advised when comparing with previous studies in which this period was 2 h (Hamvas et al., 1992; Palazzo et al., 1992). We chose this period because it is clinically representative of minor thoracic surgical procedures (Salis et al., 2008; Swanson et al., 2012) and has been used in a previous experimental study (Zhao et al., 2016). In addition, the ischemia procedure used herein only simulates the warm phase, and could not represent all events (cold and warm phases) which occur in the perioperative period of an actual transplant. Third, VV was administered with a 30% CV of V_T_ (Spieth et al., 2009), and we cannot extrapolate our findings on lung damage to other degrees of variability. Fourth, a PEEP of 2 cmH_2_O was used because, during clamp occlusion, only one lung was ventilated; together with low V_T_, this may result in lung protection (Gama de Abreu et al., 2003). Finally, although gene expression was evaluated by relative expression, previous studies (Samary et al., 2015; Spieth et al., 2015) have shown that the 2−ΔΔCt method is suitable for analysis of relative changes in gene expression in real-time quantitative PCR experiments (Schmittgen and Livak, 2008).

In conclusion, VV improved respiratory system elastance and was associated with less lung damage as compared to protective VCV in the current rat model of pulmonary ischemia-reperfusion.

## Author contributions

Conceived and designed the experiments: AS, LM, PP, PS, MG, and PR; Performed experiments: AS, LM, and RS; Analyzed data: AS, CS, RH, VC, and PS; Interpreted results of research: AS, LM, RS, CS, RH, PP, PS, MG, and PR; Drafted, edited, critically revised paper: AS, LM, RH, PP, PS, MG, and PR; All authors approved final version of manuscript.

## Funding

This work was supported by grants from the Carlos Chagas Filho Rio de Janeiro State Research Foundation (FAPERJ) [grant number E-26/103.118/2014], Rio de Janeiro, Brazil; and the Brazilian Council for Scientific and Technological Development (CNPq)/ The Department of Science and Technology (DECIT)/Brazilian Ministry of Health [grant number 469716/2014-2], Brasilia, Brazil.

### Conflict of interest statement

MG has been granted patents on variable pressure support ventilation. The other authors declare that the research was conducted in the absence of any commercial or financial relationships that could be construed as a potential conflict of interest.
