# Supplementary material for: Variable Ventilation Improved Respiratory System Mechanics and Ameliorated Pulmonary Damage in a Rat Model of Lung Ischemia-Reperfusion
Source: Front Physiol. 2017 May 2;8:257. doi: 10.3389/fphys.2017.00257 (PMC5411427; doi:10.3389/fphys.2017.00257)
Supplement: Supplementary file 1 [file Table1.DOCX]

**Supplemental Table 1. Forward and reverse oligonucleotide sequences of target gene primers**

| **Gene** | **Primer** | **Primer sequences (5′-3′)** |
| --- | --- | --- |
| IL-6 | Forward | CTC CGC AAG AGA CTT CCA G |
|  | Reverse | CTC CTC TCC GGA CTT GTG A |
| Nrf2 | Forward | GAG ACG GCC ATG ACT GAT |
|  | Reverse | GTG AGG GGA TCG ATG AGT AA |
| Ang-1 | Forward | CAGTGGCTGCAAAAACTTGA |
|  | Reverse | TCCACATCTGTGAGCTTTCG |
| Ang-2 | Forward | CAGCCAACCAGGTGATT |
|  | Reverse | AAGTTGGAAGGACCACATGC |
| Tie-2 | Forward | ATGGACTCTTTAGCCGGCTTA |
|  | Reverse | CCTTATAGCCTGTCCTCGAA |
| ICAM-1 | Forward | CTTCCGACTAGGGTCCTGAA |
|  | Reverse | CTTCAGAGGCAGGAAACAGG |
| SP-D | Forward | AAATCTTCAGGGCGGCAAA |
|  | Reverse | GGCCTGCCTGCACATCTC |
| 36B4 | Forward | AATCCTGAGCGATGTGCAG |
|  | Reverse | GCTGCCATTGTCAAACAC |

Primers used in experiments. IL-6, interleukin-6; Nrf2, nuclear factor erythroid 2-derived factor-2, Ang-1, angiopoietin-1; Ang-2, angiopoietin-2; Tie-2, angiopoietin receptor, ICAM-1, Intercellular adhesion molecule-1; SP-D, surfactant protein D; 36B4, acidic ribosomal phosphoprotein P0.
