# Supplementary material for: Variable Ventilation Improved Respiratory System Mechanics and Ameliorated Pulmonary Damage in a Rat Model of Lung Ischemia-Reperfusion
Source: Front Physiol. 2017 May 2;8:257. doi: 10.3389/fphys.2017.00257 (PMC5411427; doi:10.3389/fphys.2017.00257)
Supplement: Supplementary file 2 [file Table2.DOCX]

**Supplemental Table 2. Respiratory effects of ischemia-reperfusion injury**

| **Parameter** | **Group** | **Baseline 1** | **Baseline 2** | **Baseline 1 *vs*. Baseline 2** |
| --- | --- | --- | --- | --- |
| V_T_ (mL/kg) | Sham | 5.9 ± 0.9 | 6.0 ± 1.3 | 0.8821 |
|  | IR | 6.1 ± 0.3 | 6.1 ± 0.4 | 0.9237 |
|  |  |  |  |  |
| CV of V_T_ (%) | Sham | 2.1 ± 1.6 | 1.7 ± 0.8 | 0.3579 |
|  | IR | 1.3 ± 0.5 | 1.4 ± 1.2 | 0.6825 |
|  |  |  |  |  |
| E._RS_ (cmH_2_O/mL) | Sham | 2.3 ± 0.7 | 2.3 ± 0.5 | 0.7906 |
|  | IR | 2.3 ± 0.7 | 2.8 ± 0.5 | p=0.03 |
|  |  |  |  |  |
| E1._RS_ (cmH_2_O/mL) | Sham | 2.1 ± 0.6 | 1.8 ± 0.5 | 0.0111 |
|  | IR | 2.1 ± 0.5 | 1.4 ± 0.3 | p=0.001 |
|  |  |  |  |  |
| E2._RS_ (cmH_2_O/mL) | Sham | 0.10 ± 0.08 | 0.22 ± 0.17 | p=0.015 |
|  | IR | 0.16 ± 0.20 | 0.58 ± 0.27 | p<0.0001 |
|  |  |  |  |  |
| %E2 (%) | Sham | 10.8 ± 4.7 | 24.7 ± 14.3 | p=0.007 |
|  | IR | 15.1 ± 14.5 | 52.8 ± 12.7 | p<0.0001 |
|  |  |  |  |  |
| P_aw_ (cmH_2_O) | Sham | 10.5 ± 1.8 | 10.9 ± 1.6 | 0.6825 |
|  | IR | 11.3 ± 1.9 | 12.9 ± 2.3 | p=0.01 |
|  |  |  |  |  |
| RR (min^-1^) | Sham | 43 ± 9 | 41 ± 8 | 0.0345 |
|  | IR | 43 ± 8 | 42 ± 8 | 0.6022 |
|  |  |  |  |  |
| pHa | Sham | 7.34 ± 0.11 | 7.43 ± 0.04 | 0.9284 |
|  | IR | 7.34 ± 0.06 | 7.39 ± 0.05 | 0.1782 |
|  |  |  |  |  |
| PaO_2_/FiO_2_ | Sham | 371 ± 54 | 330 ± 51 | 0.0986 |
|  | IR | 308 ± 59 | 336 ± 68 | 0.3693 |
|  |  |  |  |  |
| PaCO_2_ (mmHg) | Sham | 34 ± 8 | 35 ± 3 | 0.6795 |
|  | IR | 36 ± 8 | 34 ± 6 | 0.3388 |
|  |  |  |  |  |
| HCO_3_^-^ (mEq/L) | Sham | 34.5 ± 10.3 | 31.8 ± 5.6 | 0.8386 |
|  | IR | 40.5 ± 8.9 | 36.8 ± 4.3 | 0.1332 |
|  |  |  |  |  |
| Fluids (mL) | Sham | 0.0 [0.0-0.0] | 0.5 [0.0-1.5] | 0.0156 |
|  | IR | 0.0 [0.0-0.0] | 0.25 [0.0-1.0] | 0.0313 |

Respiratory effects of ischemia-reperfusion injury at Baseline 1 and Baseline 2. Sham: animals subjected to surgical manipulation alone, without clamping the left pulmonary hilum; IR: animals subjected to surgical manipulation and ischemia-reperfusion injury by clamping the left pulmonary hilum. V_T_: Tidal volume; CV of V_T_: coefficient of variation of tidal volume; E,_RS_: respiratory system elastance; E1,_RS_: volume-independent elastance; E2,_RS_: volume-dependent elastance; %E2: E,_RS_ non-linearity index; P_aw_: airway pressure; pHa: arterial pH; PaO_2_/FiO_2_: arterial oxygen partial pressure divided by oxygen fraction; PaCO_2_: arterial carbon dioxide partial pressure; HCO_3_: bicarbonate; Fluids: cumulative fluids infused during the induction of ischemia-reperfusion injury. Values are mean ± standard deviation (SD) or median and interquartile range in brackets of 6 animals in each group.
