# Supplementary figures and images for: Variable Ventilation Improved Respiratory System Mechanics and Ameliorated Pulmonary Damage in a Rat Model of Lung Ischemia-Reperfusion
Source: Front Physiol. 2017 May 2;8:257. doi: 10.3389/fphys.2017.00257 (PMC5411427; doi:10.3389/fphys.2017.00257)

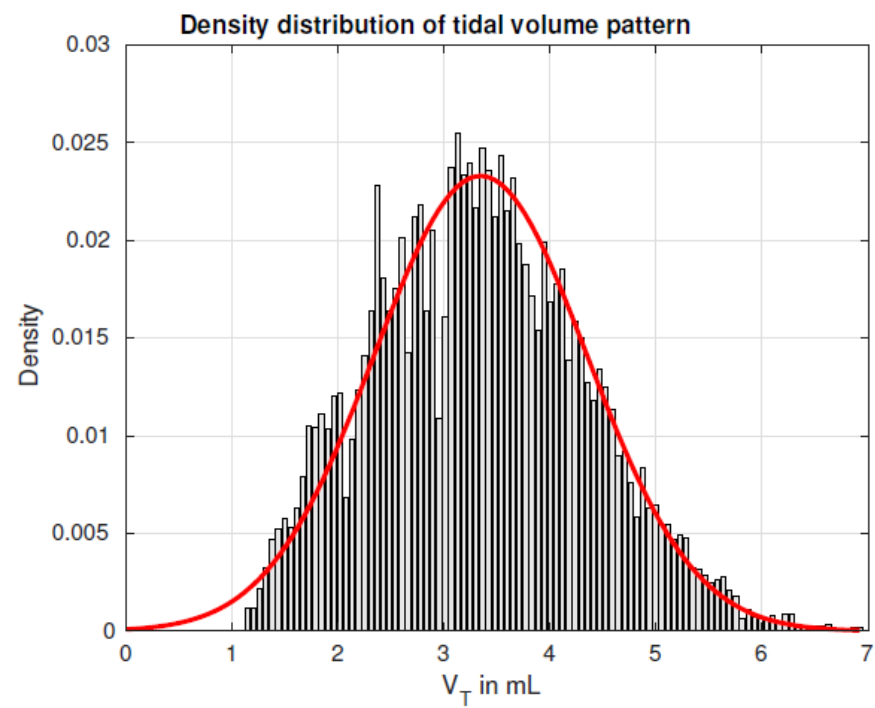

Supplemental Figure 1. Density distribution of tidal volume ( $V_T$ ) during variable ventilation.

Supplement: Supplementary file 3 [file Image1.PDF]
