# Supplementary material for: Variable Ventilation Improved Respiratory System Mechanics and Ameliorated Pulmonary Damage in a Rat Model of Lung Ischemia-Reperfusion
Source: Front Physiol. 2017 May 2;8:257. doi: 10.3389/fphys.2017.00257 (PMC5411427; doi:10.3389/fphys.2017.00257)
Supplement: Supplementary file 4 [file Image2.PDF]

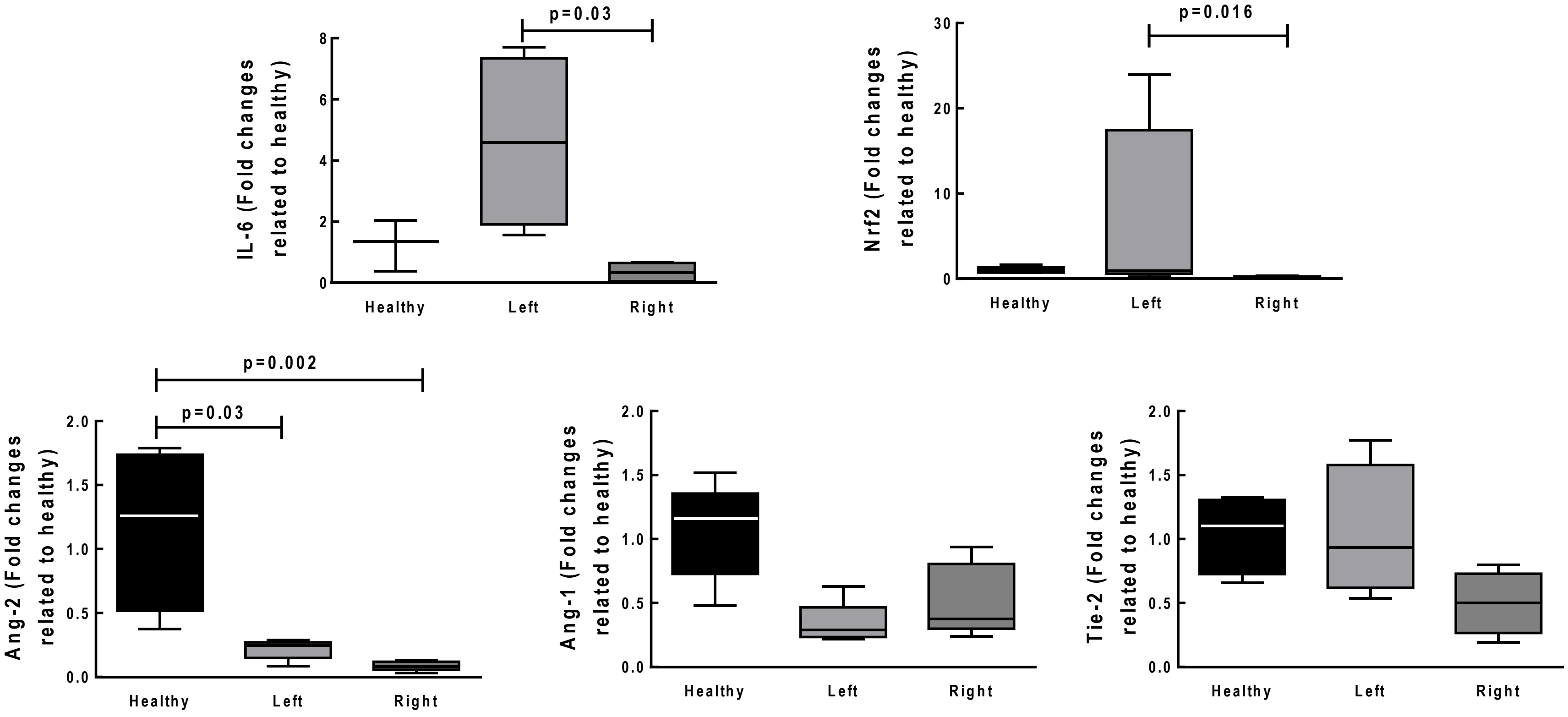

Supplemental Figure 2. Real-time polymerase chain reaction analysis of biological markers associated with inflammation [interleukin (IL)-6]; oxidative stress [nuclear factor erythroid 2-derived factor-2 (Nrf2)], and endothelial cell damage [angiopoietin (Ang) 1 and 2, receptor tyrosine kinase of Tie family (Tie), and intercellular adhesion molecular (ICAM)-1]. Left lung: ischemia-reperfusion injury; right lung: contralateral. Values represent medians and whiskers represent the 10-90 percentile range of 6 animals in each group. Relative gene expression was calculated as a ratio of the average gene expression levels compared with the reference gene (*36B4*) and expressed as fold change relative to healthy animals not subjected to the IR procedure.
